# English language learning in an international context: psychological factors among Chinese graduate students in the Philippines

**DOI:** 10.3389/fpsyg.2026.1766803

**Published:** 2026-06-01

**Authors:** Hailong Li

**Affiliations:** School of Humanities and Social Sciences, Sanya Aviation and Tourism College, Sanya, Hainan, China

**Keywords:** anxiety, Chinese graduate students, english, language acquisition, motivation

## Abstract

This study investigated the relationship between motivation and anxiety among Chinese graduate students (*N* = 300) studying in the Philippines in the post-pandemic era, a distinctive Southeast Asian English-medium instruction context, using adapted versions of the Foreign Language Classroom Anxiety Scale (FLCAS) and the Attitude/Motivation Test Battery (AMTB). The descriptive results show that both integrative motivation and instrumental motivation are high. Doctoral students are a little more motivated and a little less anxious than master's students. Nonetheless, discriminant validity assessments employing the HTMT criteria indicated that integrative motivation and fear of negative evaluations (HTMT=0.94), along with instrumental motivation and test anxiety (HTMT=0.91), cannot be empirically differentiated. These results show that there is a lot of conceptual overlap and measurement contamination. Consequently, the significant correlation observed between motivation and anxiety (ranging from 0.66 to 0.90) cannot be interpreted as indicative of a robust relationship between distinct psychological constructs. Because of this, this study should be seen as exploratory research. Its main contribution is in terms of method: it shows that you need to be careful when using Western-developed scales with Chinese students in cross-border educational settings, and it stresses how important it is to do strict discriminant validity tests. Be careful when interpreting the results of cross-cultural second language acquisition research that uses existing scales before making tools for cultural adaptation.

## 1 Introduction

As the world becomes more connected, being able to speak English well has become an important skill, especially in international academic and professional settings. English as a lingua franca (ELF) has changed how people and organizations think about learning and using English (Jenkins, 2015; Seidlhofer, 2011). In places like China, English is taught in all levels of school and is seen as necessary for academic mobility and global competitiveness (Hu, 2002; Pan and Block, 2011; Skourdoumbis and Madkur, 2020; Ullah, 2021). Because of this focus on institutions, English as a Foreign Language (EFL) programs have become more popular. Their goal is to help students become communicatively competent in a variety of language environments.

In China, English education is required from primary to tertiary levels (Wang and Phillion, 2011). However, the effectiveness of these programs is greatly affected by emotional factors, especially motivation and anxiety, which affect how engaged and well learners do (Dewaele, 2013; Zhang, 2021). The shift to English-medium instruction (EMI) overseas introduces unique psychological challenges that necessitate thorough examination. The COVID-19 pandemic has introduced additional complexities to the psychological experiences of international students. A study conducted in the Philippines during the post-pandemic transition revealed that college students exhibited moderate to high levels of depression and anxiety, with females being especially susceptible (Estacio et al., 2023). Villegas et al. (2024) also said that Filipino private university students had a lot of mental health problems during the pandemic, such as worrying about online learning and feeling alone. For Chinese graduate students who initiated or continued their studies in the Philippines during this period, these pandemic-related stressors are likely to interact with pre-existing language learning anxiety and motivation, rendering the post-pandemic context a crucial factor to consider.

Chinese students’ appetite for overseas education is growing, especially among graduate students, who see studying abroad as a way to better themselves personally and professionally. Recent data from the UNESCO Institute of Statistics (2023) shows that the Philippines remains a growing destination for Chinese graduate students, even in the post-pandemic era. This is because of its affordability and proximity as well as the long-standing partnerships it has with Chinese higher education institutions (Ramos et al., 2020). Nevertheless, as Ortiga (2026) critically observes, the internationalisation of Philippine higher education has produced complex dynamics of ethnic exclusion and social isolation for foreign students, making the sociocultural adjustment of Chinese students particularly intricate.

Such educational exchanges have opened the way for increased student and faculty mobility and have led to the development of multilingual and multicultural learning environments. In these contexts, linguistic and sociocultural adaptation is important for Chinese students to succeed academically and integrate socially. Too much anxiety can decrease participation in class, inhibit cognitive processing, and reduce working memory capacity which can all negatively affect language performance (MacIntyre and Gardner, 1991). Researchers suggest supportive classroom environments, role-play activities, formative assessment, and anxiety-aware pedagogical training for teachers to mitigate this (Cheng, 2009; Wu and Lin, 2016; Tanaka, 2023; Young, 1991).

However, the difficulties of studying in English in a foreign country are not absent. Psychological factors such as anxiety and motivation have been shown to be of key importance for the results of second language acquisition (SLA) (Horwitz, 2010; Dewaele and MacIntyre, 2014). Foreign Language Anxiety (FLA) is the anxiety that learners experience when communicating in a non-native language, which is often seen in classroom and communicative contexts (Zhang, 2021). On the other hand, motivation is the force that drives individuals to exert effort, persist and engage. It has been found to be positively correlated with language performance (Dörnyei and Ushioda, 2021; Roy and Zaman, 2017). Recent studies with structural equation modeling have shown that FLA and motivation are important predictors of success in language learning (Alqahtani, 2022; Lee, 2023; Khodadady and Khajavy, 2021).

While studies on SLA abound, studies on psychological experiences of Chinese graduate students in Southeast Asian contexts such as the Philippines are relatively scarce. Much of the existing literature has focused on western host countries, and hence has failed to account for the specific sociolinguistic and educational features of the Global South. To address this gap, this study explores the relationship between motivation and anxiety in shaping the experiences of studying the English language of Chinese graduate students in selected private higher education institutions in Metro Manila. This study sheds light on the psychological dimensions of language acquisition in a non-Western EMI setting and aims to contribute empirical insights that may lead to more inclusive and sensitive pedagogical approaches.

Theoretically motivated, the Philippines as a research site The Philippines is not like Western Anglophone countries (US, UK, Australia, etc.) in that it has a unique linguistic history as a former American colony where English has been an official language alongside Filipino for over a century, but it is not the first language for most Filipinos (Tupas, 2022). This creates what may be called a “second language” environment that is neither China’s EFL context nor native speaker-dominant Western environments. Moreover, the Philippines has some cultural similarities (collectivism, respect for hierarchy) with China that may provide some buffer to acculturation stress, compared to Western destinations.

At the same time, local varieties of English (Philippine English) vary from the standardized American or British English taught in China in terms of pronunciation, vocabulary, and discourse patterns (Tupas, 2022). These contextual features should be taken into account explicitly when interpreting Chinese students’ motivation and anxiety. Recent studies on Chinese students in study-abroad settings (Yue et al., 2022) have demonstrated that motivational dynamics are highly responsive to the characteristics of the host country, thereby justifying a focused investigation in the Philippines.

## 2 Literature review

### 2.1 Language anxiety in Chinese EFL contexts

Language anxiety is defined as “a distinct complex of self-perceptions, beliefs, feelings, and behaviors related to classroom language learning arising from the uniqueness of the language learning process” (Horwitz et al., 1986, p. 128). It is found to negatively affect learners’ performance, participation, and long-term confidence in using English (Dewaele et al., 2008; Zhang, 2022). High-stakes tests (e.g., TOEFL, IELTS), institutional pressures to publish in English, and the centrality of English for academic and professional mobility contribute to high levels of foreign language classroom anxiety among Chinese graduate students (Liu and Jackson, 2008; Wang et al., 2023).

Several sources of anxiety are particularly salient:

Communication apprehension: Fear of making mistakes or being judged by peers and instructors leads to silence and hesitancy in oral participation (Yan and Horwitz, 2008; Chen, 2023).
Test anxiety: Standardized English proficiency tests play a key role in the determination of academic and immigration outcomes, which can be very stressful psychologically (Zheng, 2008; Kim and Kim, 2024). Adding to these traditional causes of anxiety were new stressors brought on by the COVID-19 pandemic. Studies in the Philippines have shown that college students transitioning after the pandemic reported moderate anxiety, and online learning environments increased their feelings of isolation and academic stress (Estacio et al., 2023; Villegas et al., 2024). For Chinese graduate students, the transition to hybrid or fully online instruction may have exacerbated test anxiety due to the novelty of the assessment formats and lack of social support.
Fear of negative evaluation: Students fear being perceived as incompetent, particularly in competitive academic settings where peer comparison is prevalent (Liu, 2006; Wang and Lee, 2022).

High levels of anxiety can lead to decreased classroom participation, impaired cognitive processing, and reduced working memory capacity, which in turn negatively affect language performance (MacIntyre and Gardner, 1991). In order to mitigate this, researchers recommend supportive classroom environments, role-play activities, formative assessment and anxiety-aware pedagogical training for teachers (Cheng, 2009; Wu and Lin, 2016; Tanaka, 2023).

### 2.2 The role of motivation in language learning

Another basic factor in SLA is motivation. Important frameworks such as the socio-educational model by Gardner (1985), Self-Determination Theory (SDT) by Deci and Ryan (1985) and L2 Motivational Self System by Dörnyei (2005, 2009) provide insight into motivational orientations. Gardner (1985) differentiated between integrative motivation (interest in the language and culture) and instrumental motivation (utilitarian goals including job or academic success). Motivation is viewed by SDT as a continuum between intrinsic (internal motivation) and extrinsic (external motivation). Dörnyei’s model further contextualizes motivation through the Ideal L2 Self (aspirations), Ought-to L2 Self (external pressures) and the L2 Learning Experience (immediate learning conditions). The L2 Motivational Self System has been used in a variety of cultural contexts, including Chinese learners (Taguchi et al., 2009).

Chinese graduate students are often highly instrumentally motivated as English is seen as important for social mobility and academic prestige (Gao, 2010; Wen and Clément, 2003; Liu, 2023). Gao et al. (2007) found that intrinsic motivation was less common, but it was present among students who had a genuine interest in English speaking cultures. Studies have found that students who are intrinsically motivated are more likely to attain higher proficiency and remain engaged for longer periods of time (Deci and Ryan, 1985; Ushioda, 2020; Ryan and Deci, 2020). This understanding has been broadened by recent research on Chinese students abroad. According to Yue et al. (2022), for Chinese postgraduate students in the UK, “international posture” and the “ideal L2 self” were the strongest positive predictors of motivated learning behavior, while the “ought-to L2 self” negatively affected this behavior, which suggests that the external pressure may backfire in study-abroad contexts. This finding complicates the simple dichotomy of instrumental vs. integrative motivation and calls for context-sensitive investigations.

### 2.3 The interplay between anxiety and motivation

The link between anxiety and motivation is dynamic and complex. While motivation can serve as a buffer to the negative effects of anxiety, high levels of anxiety tend to have a detrimental effect on learners’ motivation and confidence, and create avoidance behavior (Dörnyei, 2005; Zhang, 2022). For example, students may experience performance anxiety when their academic aspirations are extremely high, which is especially true for highly motivated students (Liu and Huang, 2011; Chen and Zhang, 2024). Studies also find that students with instrumental motivation report higher anxiety, because of the pressure to fulfill external expectations (Wen and Clément, 2003), while integrative motivation is related to more relaxed engagement and less anxiety (Gardner and Lambert, 1972; Park, 2023).

This two-way relationship must be addressed. Targeted motivational strategies such as personalized goal-setting, culturally relevant pedagogy, and reflective practices that create a low-anxiety learning environment can improve psychological well-being and academic success (Cheng and Dörnyei, 2007; Gu, 2009; Nguyen, 2024). Bandura’s (1997) self-efficacy theory posits that mastery experiences build confidence and reduce anxiety.

### 2.4 The Philippine context and research gap

While extensive research has examined the interplay of anxiety and motivation in Western EFL contexts (e.g., Dewaele and MacIntyre, 2014) or within mainland China (Liu and Huang, 2011), there is a notable scarcity of empirical inquiry into the psychological experiences of Chinese graduate students in Southeast Asian host countries. The Philippines presents a unique “Global South” EMI context, distinct from Western Anglophone nations, where sociocultural adjustment and academic expectations may differentially shape affective variables. Factors such as the Philippines’ colonial history with English, its role as a major English-speaking nation in Asia, and the growing bilateral educational agreements with China create a distinctive learning ecology (Ramos et al., 2020; Tupas, 2022).

Recent empirical work has begun to document the specific challenges faced by international students in the Philippines. Ortiga (2026) showed that private universities’ aggressive recruitment of international students led to backlash among Filipino educators and students, resulting in policies that isolated foreign students on campus. Such ethnic exclusion and racism, as Ortiga terms it, can profoundly affect international students’ sense of belonging and, by extension, their language learning motivation and anxiety.

Meanwhile, comparative research on Chinese students’ anxiety and motivation has revealed that international students exhibit distinct patterns of communication apprehension and acculturation stress compared to their domestic peers (Luo, 2025). Ding et al. (2025) found that for Chinese EFL learners, oral presentation task anxiety was significantly negatively correlated with performance, but not with enjoyment, highlighting the importance of considering specific task types and contexts.

Despite these advances, no study has yet systematically investigated the psychometric properties of standard FLCAS and AMTB scales among Chinese graduate students in the Philippines. Neither has any study examined whether the constructs of motivation and anxiety are empirically distinct among this population. This study fills this gap by investigating the expression of these psychological constructs, and the validity of their measurement, in this under-researched transnational context. Following the principles of a growth mindset (Dweck, 2006), focusing on progress via effort, not performance via outcome, can prevent motivation from being self-imposed pressure.

## 3 Problem statements

It is generally accepted that anxiety and motivation are important psychological constructs that affect the outcomes of L2 learning, especially in EFL contexts. These factors are even more vital for Chinese graduate students studying abroad, since they have to adapt to linguistic, cultural and academic differences in English-medium institutions. Higher motivation can assist in engaging with the language and learning it whereas higher anxiety can negatively affect communicative performance and cognitive processing and in turn, learning outcomes (Horwitz et al., 1986; Dörnyei, 2005; Dewaele and MacIntyre, 2014).

As more Chinese students pursue graduate degrees in the Philippines and as they are exposed to a unique sociolinguistic environment, it is important for educators, language teachers, and policymakers to understand how these affective variables interact. Understanding learners’ emotional and motivational profiles is key to designing pedagogically responsive language programs that support academic achievement and psychological well-being.

Given these theoretical and practical considerations, the present study has sought to examine the psychological dimensions of English language acquisition among Chinese graduate students in the context of the Philippines by answering the following research questions:

What is the foreign language anxiety level of Chinese graduate students studying in Philippine higher education institutions?
What is the level of motivation of Chinese graduate students in learning English in a transnational academic context?
What is the statistical significance of the relationship between anxiety and motivation in Chinese graduate students’ English language learning experiences?

## 4 Methods

### 4.1 Research design

The quantitative research design was used in this study, specifically the descriptive-correlational method to determine the levels of language learning anxiety and motivation among Chinese graduate students and to find out the relationship between these two psychological constructs. Quantitative research involves the collection and statistical analysis of numerical data to describe variables and test hypotheses about relationships among variables (Creswell and Creswell, 2018; McMillan and Schumacher, 2010). The descriptive-correlational design was appropriate for this research as it enables finding patterns, relationships, and predictive associations, without manipulating the research variables (Ary et al., 2019).

### 4.2 Participants

A total of 300 Chinese graduate students enrolled in master’s and doctoral programs from three private universities in the National Capital Region (NCR) of the Philippines were recruited using random sampling techniques to ensure representativeness and reduce sampling bias. Demographic characteristics of the participants are shown in Table 1.

**Table 1:** Demographic characteristics of participants (*N* = 300).

Characteristic	Category	*n*	%
Degree program	MA	165	55.0
	PhD	135	45.0
Gender	Male	128	42.7
	Female	172	57.3
Length of stay in PH	< 1 year	89	29.7
	1–2 years	142	47.3
	> 2 years	69	23.0
Field of study	Humanities/Social Sciences	112	37.3
	STEM	98	32.7
	Business/Management	90	30.0

### 4.3 Instrumentation

Data were collected using a two-part survey instrument. All items were rated on a 5-point Likert scale ranging from 1 (Strongly Disagree) to 5 (Strongly Agree).

#### 4.3.1 Part 1: motivation scale

This section measured language learning motivation using a 20-item scale adapted from Gardner (1985) Attitude/Motivation Test Battery (AMTB), comprising two subscales: Integrative Motivation (10 items; e.g., “Learning English helps me understand English-speaking cultures better”) and Instrumental Motivation (10 items; e.g., “English will be useful for my future career”).

#### 4.3.2 Part 2: anxiety scale

This section measured foreign language anxiety using a 15-item scale adapted from Horwitz et al.'s (1986) Foreign Language Classroom Anxiety Scale (FLCAS), comprising three subscales: Communication Apprehension (5 items; e.g., “I feel nervous speaking English in class”), Test Anxiety (5 items; e.g., “I worry about failing English tests”), and Fear of Negative Evaluation (5 items; e.g., “I am afraid others will laugh at me when I speak English”).

### 4.4 Validity and reliability

The instrument underwent content validation by three field experts in applied linguistics and educational psychology. Pilot testing was conducted with 30 Chinese graduate students (not included in the main sample), followed by reliability analysis using Cronbach’s alpha to ensure internal consistency of subscales.

**Table:** 

Motivation	Number of Items	Cronbach’s alpha
Integrative motivation	10	0.876
Instrumental motivation	10	0.782
All	20	0.823

**Table:** 

Anxiety	Number of items	Cronbach’s alpha
Communication apprehension	5	0.701
Test anxiety	5	0.818
Fear of negative evaluation	5	0.794
All	15	0.771

### 4.5 Data collection and analysis

The data were collected through online and paper-based surveys administered during the 2024–2025 academic year. The data collection period (2024–2025) coincided with the post-pandemic transition in the Philippines, when most universities had resumed in-person or hybrid instruction. This context may have influenced students’ psychological states, as pandemic-related stressors were still present (Estacio et al., 2023; Villegas et al., 2024). All responses were coded and analyzed using the Statistical Package for the Social Sciences (SPSS) version 28.

Descriptive statistics (means, standard deviations) were computed to determine the general levels of anxiety and motivation (Research Questions 1 and 2). Pearson’s correlation coefficient was calculated to assess the strength and direction of the relationship between the two constructs (Research Question 3). Inferential statistics were employed to test the stated hypotheses at a 0.05 level of significance.

### 4.6 Common method bias

Since all data were collected via self-report questionnaires, there is a potential risk of common method bias (CMB). To examine the possible influence of CMB, we conducted Harman’s single-factor test (Podsakoff et al., 2003). An unrotated exploratory factor analysis was performed on all measurement items. The results showed that the first factor accounted for 32.4% of the total variance, which is below the 50% threshold, indicating that common method bias does not pose a serious threat to the validity of the findings.

## 5 Results

### 5.1 Descriptive statistics: levels of motivation and anxiety

Table 2 presents the descriptive statistics for motivation and anxiety subscales, disaggregated by degree program (MA vs. PhD).

**Table 2:** Descriptive statistics for motivation and anxiety by group.

Variable	Group	Mean	SD	Interpretation
Integrative motivation	MA students	3.85	0.52	High
	PhD students	4.12	0.48	High
Instrumental motivation	MA students	4.02	0.55	High
	PhD students	4.28	0.50	High
Communication apprehension	MA students	3.45	0.60	Moderate
	PhD students	2.98	0.58	Moderate
Test anxiety	MA students	3.22	0.62	Moderate
	PhD students	3.10	0.59	Moderate
Fear of negative evaluation	MA students	3.60	0.65	Moderate/High
	PhD students	3.05	0.61	Moderate

Interpretation based on scale: 1.00–2.49 = Low; 2.50–3.49 = Moderate; 3.50–5.00 = High.

The results show that both MA and PhD student groups reported high levels of integrative and instrumental motivation. PhD students scored slightly higher on both types of motivation. In contrast, MA students reported higher levels of all three anxiety subtypes (especially fear of negative evaluation) than their PhD counterparts.

### 5.2 Correlations between motivation and anxiety

Table 3 presents the Pearson correlation coefficients between the two motivational constructs and three anxiety subtypes.

**Table 3:** Correlation between motivation and anxiety (*N* = 300).

Variables	Integrative Motivation	Instrumental Motivation
Communication apprehension		
Pearson Correlation	0.729**	0.663**
Sig. (2-tailed)	<0.001	<0.001
Test anxiety		
Pearson Correlation	0.864**	0.865**
Sig. (2-tailed)	<0.001	<0.001
Fear of negative evaluation		
Pearson Correlation	0.901**	0.799**
Sig. (2-tailed)	<0.001	<0.001

**p < 0.01 (2-tailed).

The results show that there are consistently strong positive correlations between both types of motivation and all three types of anxiety. The coefficients range from moderate (r = 0.663) to very strong (r = 0.901). The most significant correlation identified was between integrative motivation and fear of negative evaluation (r = 0.901, *p* < 0.001).

### 5.3 Confirmatory factor analysis (CFA)

The factor structure and the discriminant validity of the constructs were tested using confirmatory factor analysis (CFA) with AMOS 28. Two models were compared:

Model 1 (One-factor model): All items loaded on one factor.

Model 2 (Five-factor model): Items loaded on five theoretically distinct factors, namely, Integrative Motivation, Instrumental Motivation, Communication Apprehension, Test Anxiety, and Fear of Negative Evaluation.

The results show that the five-factor model significantly outperformed the one-factor model across all fit indices and met the recommended criteria for good model fit (χ^2^/df = 2.31; CFI = 0.92; RMSEA = 0.058). This indicates that participants were able to distinguish among the five theoretical constructs, supporting the factor structure validity of the scales.

### 5.4 Discriminant validity

To further assess discriminant validity, we applied the Fornell and Larcker (1981) criterion. This approach compares the square root of the average variance extracted (AVE) for each construct with the correlations between that construct and others. Discriminant validity is considered adequate if the square root of AVE is larger than the corresponding inter-construct correlations.

The findings demonstrate:

All constructs exhibited AVE values exceeding 0.50 (ranging from 0.55 to 0.67), signifying satisfactory convergent validity.

For the majority of construct pairs, the square root of AVE surpassed the inter-construct correlations, thereby affirming discriminant validity.

The correlation between Integrative Motivation and Fear of Negative Evaluation (r = 0.90) was marginally superior to their respective AVE square roots (0.82 vs. 0.78). This indicates that although these two constructs are statistically distinguishable, they possess significant conceptual overlap. This finding is consistent with the theoretical interpretation of the “Anxiety-Motivation Complex” previously articulated in the Discussion, indicating that for learners from this cultural background, motivation and anxiety are psychologically interconnected experiences.

To provide a more rigorous test of discriminant validity, we calculated the Heterotrait-Monotrait (HTMT) ratio of correlations (Henseler et al., 2015). The HTMT criterion is considered more stringent than the Fornell-Larcker approach; values below 0.85 or 0.90 indicate adequate discriminant validity.

The HTMT results in Table 4 show that the values for integrative motivation with fear of negative evaluation (0.94) and instrumental motivation with test anxiety (0.91) are above the threshold value of 0.90. This indicates that these construct pairs lack discriminant validity as per the guidelines (Henseler et al., 2015). Therefore, there is no empirical basis for any substantive interpretation of the correlations between these pairs of constructs. The high correlations reported in Table 3 (e.g., r = 0.901) are most likely a methodological artifact of construct overlap rather than evidence of a true relationship between separate psychological variables (Table 5).

**Table 4:** Confirmatory factor analysis: model fit indices.

Model	χ^2^/df	CFI	TLI	RMSEA	SRMR
Model 1 (One-factor)	4.82	0.67	0.64	0.113	0.102
Model 2 (Five-factor)	**2.31**	**0.92**	**0.91**	**0.058**	**0.049**
Recommended cutoffs	< 3.0	> 0.90	> 0.90	< 0.08	< 0.08

Bold values indicate HTMT ratios exceeding the 0.90 threshold, suggesting lack of discriminant validity.

**Table 5:** Discriminant validity: AVE square roots vs. correlations.

Construct	1	2	3	4	5
1. Integrative motivation	**(0.82)**				
2. Instrumental motivation	0.76	**(0.79)**			
3. Communication apprehension	0.73	0.66	**(0.74)**		
4. Test anxiety	0.86	0.87	0.71	**(0.80)**	
5. Fear of negative evaluation	0.90	0.80	0.75	0.82	**(0.78)**

Diagonal values in bold represent the square root of AVE; off-diagonal values represent inter-construct correlations.

A careful reader might see a conflict between the five-factor CFA model’s good fit (CFI = 0.92, RMSEA = 0.058) and the HTMT ratios’ lack of discriminant validity (e.g., 0.94). This apparent contradiction can be clarified as follows.

Global fit indices like CFI and RMSEA look at how well the hypothesized factor structure matches the observed covariance matrix as a whole. A model can attain satisfactory global fit despite certain pairs of constructs demonstrating elevated inter-factor correlations, provided that the overall configuration of loadings and covariances aligns closely with the designated structure (Fornell and Larcker, 1981; Henseler et al., 2015). The five-factor model fits better than a one-factor model (Table 6), but this does not mean that all construct pairs are different enough.

**Table 6:** HTMT ratios for construct pairs.

Construct pair	HTMT ratio
Integrative motivation ↔ Instrumental motivation	0.79
Integrative motivation ↔ Communication apprehension	0.81
Integrative motivation ↔ Test anxiety	0.89
Integrative motivation ↔ Fear of negative evaluation	**0.94**
Instrumental motivation ↔ Communication apprehension	0.74
Instrumental motivation ↔ Test anxiety	0.91
Instrumental motivation ↔ Fear of negative evaluation	0.85

Second, HTMT is specifically developed to detect the lack of discriminant validity and it is more sensitive to high correlations between constructs than global fit indices (Henseler et al., 2015). A HTMT value greater than 0.90 suggests that the average correlation between items of different constructs is almost as high as the average correlation among items within the same construct. If the high correlation is between only a subset of construct pairs, as here (integrative motivation with fear of negative evaluation; instrumental motivation with test anxiety), this condition can be met with an acceptable global fit.

Third, the current findings illustrate a case where a theoretically plausible five-factor structure is supported by global fit indices but specific pairs of constructs do not meet the more rigorous criteria of discriminant validity. This highlights the need to complement CFA with dedicated tests for discriminant validity such as HTMT, particularly in cross-cultural research where construct boundaries may become blurred due to cultural or linguistic factors.

Therefore, the good overall fit of the five-factor model does not contradict the conclusion that in this sample integrative motivation and fear of negative evaluation (and also instrumental motivation and test anxiety) are empirically indistinguishable. The main methodological conclusion remains valid: the scales do not sufficiently discriminate between these constructs in this population, and substantive interpretations of correlations are not justified.

## 6 Discussion

This study investigates the motivation and anxiety levels of Chinese graduate students in the Philippines. The descriptive results in Table 2 indicated that the students in both the MA and PhD groups reported high levels of integrative and instrumental motivation, consistent with the previous studies of Chinese learners (Gao, 2010; Wen and Clément, 2003). PhD students scored a little higher on motivation and lower on all three types of anxiety than MA students. This pattern may be attributed to the longer exposure of PhD students to English-medium academic tasks and their higher linguistic self-efficacy (MacIntyre and Gardner, 1991; Dewaele, 2013).

But the main finding of this study is methodological rather than substantive. The HTMT discriminant validity analysis indicated that two pairs of constructs, Integrative motivation and fear of negative evaluation, and Instrumental motivation and test anxiety, were empirically indistinguishable. That is, the tests designed to measure these distinct constructs did not perform as expected for this particular sample of Chinese graduate students.

What happened to the scales? One explanation may be cultural. In Confucian heritage cultures, academic striving and fear of social evaluation are often deeply interwoven (Li J., 2022; Li W., 2022; Wen and Clément, 2003). A highly motivated student to learn English may also have a lot anxiety about losing face when speaking in public. The original scales, developed in Western contexts, may assume a cleaner separation between these affective dimensions than is true for this population. Another possibility is that translation and adaptation may have introduced semantic shifts that blurred construct boundaries. These explanations are speculative and need empirical testing. Recent work by Wang et al. (2024) also underscores the importance of self-regulated learning in multilingual contexts. The measurement issues discussed here relate to the broader issues of cross-cultural SLA research. Yue et al. (2022) found that the “ought-to L2 self” — a construct related to external pressures — had a negative impact on Chinese students’ motivation in the UK, suggesting that the relationship between motivational and affective variables is highly context-dependent. In the Philippine context, the analysis of ethnic exclusion by Ortiga (2026) implies that international students may experience increased social evaluative threat, which may obscure the conceptual boundary between wanting to succeed (motivation) and fearing negative judgment (anxiety). Moreover, Zhang (2025) argued that the interaction between internal and external factors affecting the academic values of graduate students is complex, and the failure of simple dichotomies to accurately describe lived realities.

Taken together, these studies support the interpretation that the FLCAS and AMTB may not adequately separate motivation and anxiety for Chinese learners in the Philippines.

Thus, the correlations shown in Table 3 (e.g., r = 0.901 between integrative motivation and fear of negative evaluation) cannot be taken as evidence for any meaningful relation between different psychological constructs. The most probable explanation is measurement contamination. The present study does not purport to determine whether or not motivation and anxiety are positively or negatively correlated in this population. The only conclusion that can be supported is that the instruments in use are not discriminant valid for Chinese graduate students studying in the Philippines.

## 7 Limitations

The main weakness of this study is the empirical indistinguishability of key construct pairs as argued above, this renders any substantive claims about the motivation-anxiety relation invalid. Thus, the results should be considered exploratory and hypothesis-generating rather than confirmatory. The main contribution of this study is to identify a measurement problem that needs to be resolved before meaningful investigation of the motivation-anxiety relationship can be carried out.

Other limitations include the correlational nature of the design (i.e., no causal inferences), use of self-report data (i.e., possible response bias), and the cross-sectional nature of the design (i.e., only a snapshot). Future studies should develop and validate culturally adapted instruments and use qualitative methods to generate items that represent how Chinese learners conceptualize motivation and anxiety themselves. Future research would also benefit from mixed-methods and longitudinal designs. Moreover, the data were collected during the post-pandemic transition (2024–2025), a time when pandemic-related disruptions in learning and mental health were still apparent (Estacio et al., 2023). Findings may not be generalizable to pre-pandemic or fully recovered contexts.

This pattern may reflect PhD students’ longer exposure to English-medium academic tasks and greater linguistic self-efficacy (MacIntyre and Gardner, 1991, 1994; Dewaele, 2013). Similar findings have been reported among doctoral students in other EMI contexts (Kim, 2023).

## 8 Methodological implications

The findings have implications for how researchers use existing scales in cross-cultural SLA research. First, discriminant validity testing (e.g., HTMT) should be a standard reporting practice when adapting established instruments to new populations. Second, scale adaptation requires more than translation; it should involve qualitative exploration of construct meanings and empirical validation of factor structures. Third, when discriminant validity is lacking, researchers should refrain from substantive interpretation of correlations between those constructs. The appropriate response is to acknowledge the measurement problem and focus on methodological contributions.

## 9 Conclusion

This study set out to examine the relationship between motivation and anxiety among Chinese graduate students in the Philippines. However, discriminant validity testing showed that integrative motivation was empirically indistinguishable from fear of negative evaluation, and instrumental motivation was indistinguishable from test anxiety. Therefore, the observed high correlations cannot be interpreted as evidence of a relationship between distinct constructs. The study is exploratory, and its primary contribution is methodological: it demonstrates the need for caution when applying Western-developed scales to Chinese learners in transnational settings. Future research must prioritize instrument development and validation before any claims about the motivation-anxiety relationship can be made. Given the post-pandemic timing of data collection, future longitudinal research should examine whether the discriminant validity of these instruments improves as educational and social conditions stabilize, or whether fundamental cultural-linguistic mismatches persist.

## Data Availability

The datasets presented in this study can be found in online repositories. The names of the repository/repositories and accession number(s) can be found in the article/supplementary material.
